# Single-vehicle and Multi-vehicle Accidents Involving Motorcycles in a Small City in China: Characteristics and Injury Patterns

**DOI:** 10.3934/publichealth.2015.1.74

**Published:** 2015-03-19

**Authors:** Lili Xiong, Liping Li

**Affiliations:** 1 Injury Prevention Research Center, Medical College of Shantou University, 22 XinLing Road, Shantou 515041, China; 2 Maternal and Children Health Care Hospital of Hunan Province, 53 Xiangchun Road, Changsha 410000, China

**Keywords:** motorcycle, injuries, accident types, injury patterns, mortality

## Abstract

**Introduction:**

There is a gap that involves examining differences between patients in single-vehicle (SV) versus multi-vehicle (MV) accidents involving motorcycles in Shantou, China, regarding the injury patterns and mortality the patients sustained. This study aims to address this gap and provide a basis and reference for motorcycle injury prevention.

**Method:**

Medical record data was collected between October 2002 and June 2012 on all motorcycle injury patients admitted to a hospital in the city of Shantou of the east Guangdong province in China. Comparative analysis was conducted between patients in SV accidents and patients in MV accidents regarding demographic and clinic characteristics, mortality, and injury patterns.

**Results:**

Approximately 48% (*n* = 1977) of patients were involved in SV accidents and 52% (*n* = 2119) were involved in MV accidents. The average age was 34 years. Collision of a motorcycle with a heavy vehicle/bus (4%) was associated with a 34 times greater risk of death (RR: 34.32; 95% CI: 17.43–67.57). Compared to patients involved in MV accidents, those involved in SV accidents were more likely to sustain a skull fracture (RR: 1.47; 95% CI: 1.22–1.77), an open head wound (RR: 1.46; 95% CI: 1.23–1.74), an intracranial injury (RR: 1.39; 95% CI: 1.26–1.53), a superficial head injury (RR: 1.37; 95% CI: 1.01–1.86), an injury to an organ (RR: 2.01; 95% CI: 1.24–3.26), and a crushing injury (RR: 1.98; 95% CI: 1.06–3.70) to the thorax or abdomen. However, they were less likely to sustain a spinal fracture (RR: 0.58; 95% CI: 0.39–0.85), a pelvic fracture (RR: 0.22; 95% CI: 0.11–0.46), an upper extremity fracture (RR: 0.75; 95% CI: 0.59–0.96), or injuries to their lower extremities, except for a dislocation, sprain, or injury to a joint or ligament (RR: 0.82; 95% CI: 0.49–1.36).

**Conclusion:**

The relative risk of death is higher for patients involved in multi-vehicle accidents than patients in single-vehicle accidents, especially when a collision involves mass vehicle(s). Injury to the head dominated motorcycle injuries. Single-vehicle accidents have a higher correlation with head injury or internal injuries to the thorax or abdomen. Multi-vehicle accidents are more correlated with extremity injuries, especially to the lower extremities or external trauma to the thorax or abdomen.

## 1. Introduction

Compared to traditional automobiles, motorcycles are easier to maneuver, consume less fuel, and have shorter acceleration and transit times [1]. Motorcycles have become a popular means of transportation worldwide, especially in middle and low income countries. Motorcycle related injuries have become a major public health problem. Motorcycle accidents are sometimes multi-vehicle (MV), in addition to a motorcycle. Other times the accidents involve just one motorcycle, and are therefore single-vehicle (SV). By taking the running distance as the exposure measurement for risk, motorcyclists had approximately 3.9 times greater risk of involvement in a single-vehicle fatal accident compared with non-motorcycle drivers [2]. Hurt *et al* showed that the likelihood of injury in a motorcycle accident was 98% in MV collisions and 96% in SV accidents, with 55% of all accidents resulting in moderate or severe injuries [1].

SV and MV accidents have different mechanisms of occurrence and critical risk factors (e.g., age, collision type, helmet use, unsafe speed) [3],[4]. Prediction statistics analysis models constitute the primary research focus in order to find the risk factors related to human-vehicle-environment-traffic system to explore the differences between SV and MV accident types in motorcycle injuries [5]–[9]. There are clear appropriate countermeasures that can be implemented for primary injury prevention, as previous research has provided the relative effect of various risk factors in both SV and MV accidents. However, it is unclear what effect secondary prevention has on lessening patient mortality when presented to a hospital, as 34–50% of trauma deaths occur in hospitals [10]. These deaths could possibly be prevented by optimization of trauma care. Few studies have compared the characteristics of victims involved in SV accidents and those in MV accidents in motorcycle injuries.

This study is important due to the literary gap concerning the examination of differences between patients in SV and patients in MV motorcycle injuries in China and regarding the injury patterns and mortality sustained by the patients. This study aims to address this gap in the literature by reviewing medical charts to compare demographic and clinic characteristics, mortality, and injury patterns of patients involved in SV and MV accidents, in addition to providing a basis and reference for motorcycle injuries prevention.

## 2. Materials and Method

We performed a prospectively collection of the hospital medical data of patients admitted to the university hospital affiliated with the Shantou Medical College in Shantou following motorcycle injuries from 1 October 2002 to 1 June 2012. The study was approved by the institutional ethics review board of Shantou Medical College. All medical records were anonymous and the information extracted was only used for research purposes. A motorcycle injury was defined as any injury resulting from a motorcycle traffic accident regardless of the severity or outcome. Cases involving a motorcycle in a road traffic injury were extracted according to the international statistical classification of diseases and related health problems Version 10(ICD-10) [11]. According to the definition of injury severity, all objects in the study were classified as serious injury, meaning the casualty was detained in a hospital as an inpatient for more than 12 subsequent hours. Study personnel divided cases into SV and MV accidents. A SV accident type refers to an accident that does not involve other moving objects [12]. From the clinic notes, the pedestrians' status could not be judged, so collisions involving pedestrian(s) were grouped into SV. A MV accident type refers to an accident that involves at least two vehicles, one of them being a motorcycle. The following variables were extracted from clinic notes: gender, age, marriage status, status in road traffic, time of admission, duration of hospital stay, total medical costs for the relevant hospitalization, and main disease diagnosis. Injuries were further classified by the main disease diagnosis according to broad ICD-10 classifications. For example, injuries of the thorax or abdomen were combined to divide into open wounds, crush, and so on for ease of analysis and presentation.

The data were analyzed by using STATA 12.0. Differences in demographic and clinic properties were tested using a χ^2^ test for categorical variables and nonparametric Mann-Whitney U-test for continuous variables after checking for normality using Skewness/Kurtosis tests. Mortality and relative risk (RR) ratios with 95% confidence intervals (CI) for different collision types in SV and MV were computed. Injury patterns and relative risk (RR) ratios with 95% confidence intervals (CI) for SV and MV accidents were computed and statistical significance was set at *p* < 0.05.

## 3. Results

**Figure 1.**
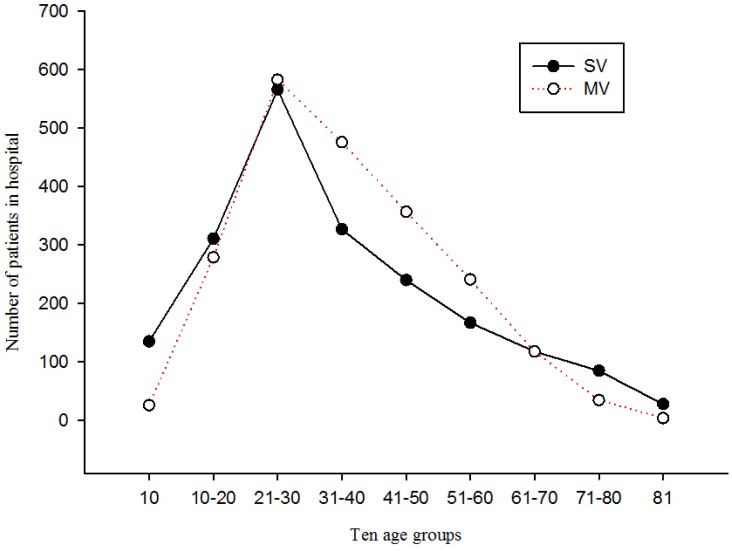
The number of patients in hospital among age groups.

During the study period, 4,096 patients required hospitalization due to an injury sustained in a motorcycle injury. There were 1,977 (48.27%) cases involved in SV and 2,119 (51.73%) cases involved in MV. Information of 17 patients was missing, excluding them from the analysis of injury patterns. Males accounted for 2,984 (72.85%) of the patients. The ratio of males to females was 2.68:1. The age ranged from 1 to 93 years, with a mean age of 34.39 (SD = 16.76) years and the majority (50%) of cases were aged 22–45. Age was stratified into ten year blocks in the two accident types. The average age was 33.60 (SD = 0.42) years in SV accidents and 35.13 (SD = 0.33) years in MV accidents. The age distribution of the total patients determined to be normal after normality test (Z = −5.00, *p* = 0.00). However, the number of patients in the 21-70 age groups in MV accidents was greater than those in SV (Figure 1). Most patients were admitted for motorcycle injuries during the months of October (*n* = 380, 9.28%), November (*n* = 367, 8.96%), February (*n* = 362, 8.84%), and March (*n* = 360, 8.79%), although there was no difference in the proportion of the number of inpatients in the two accident types between the months (*χ*^2^(11) = 16.84, *p* = 0.12).

### 3.1. Accident types and relative risk for mortality

As shown in Table 1, the collision of a motorcycle with a car or a truck (*n* = 1147; 28%) was the most common collision type, followed by collision of a motorcycle with barrier (*n* = 888; 21.68%), collision of a motorcycle with a pedestrian (*n* = 519; 12.67%), and collision of a motorcycle with a motorcycle (*n* = 490; 11.96%). The total mortality in-hospital for patients involved in motorcycle injuries was 76 (1.86%), with 28 (1.42%) individuals involved in SV accidents and 48 (2.18%) involved in MV accidents. There was difference in the proportion of SV and MV accidents between the dead and the survivors (*χ*^2^(1) = 4.05, *p* = 0.04). Risk of death was higher for collisions of a motorcycle with a heavy vehicle/bus (RR: 34.32; 95% CI: 17.43–67.57) and collisions of a motorcycle with an “undefined” object in MV accidents (RR: 3.34; 95% CI: 1.26–8.88).

**Table 1.** Accident types and relative risk for mortality.

Collision Types		Total (4096, %)	Survivor	Mortality	RR	95% CI
Single vehicleaccidenttype	Barrier	888 (21.68)	881	7	0.37	0.17–0.79
	Pedestrian	519 (12.67)	509	10	1.04	0.54–2.02
	Fixed object	501 (12.23)	492	9	0.96	0.48–1.92
	Other	69 (1.68)	67	2	1.58	0.40–6.30
Multiple vehicles accidenttype	Car/truck	1147(28.00)	1129	18	0.80	0.47–1.35
	Motorcycle	490 (11.96)	419	11	1.44	0.77–2.71
	Cyclist	202 (4.93)	197	5	1.36	0.55–3.32
	Heavy	152 (3.71)	143	9	34.32	17.43–67.57
	vehicle/bus					
	Other	67 (1.64)	63	4	3.34	1.26–8.88
	Mo-ped	61 (1.49)	60	1	0.89	0.12–6.24
	/scooter					

*The collision type is a collision of a motorcycle with the indicated vehicle.

### 3.2. Comparison between SV and MV accident types regarding demographic and clinic characteristics

With regard to marriage status, the proportion of marriage was higher for patients in SV accidents (*n* = 1,060, 53.62%) than patients in MV accidents (*n* = 1,366, 64.46%; *χ*^2^(1)= 49.84, *p* = 0.00) in Table 2. There were fewer passengers (*n* = 1,222, 61.81%) in SV than passengers (*n*= 1,634, 77.11%) in MV accidents and more riders (*n* = 282, 14.26%) in SV than riders in MV (*n* = 287, 13.54%). The total number of pedestrians and cyclists were 470 (11.47%) and 193 (4.71%), with the remaining 8 patients undefined. With regard to admission time, weekends (Friday to Sunday) were significantly less frequent in the MV accidents (*n* = 858, 40.49%) compared with SV accidents (*n* = 867, 43.85%; *χ*^2^(1) = 4.75, *p* = 0.03). Furthermore, there was difference in the proportion of the origin of the patients in the two accident types (*χ*^2^(3) = 48.19, *p* = 0.00). Victims in SV accidents were more likely than patients involved in MV accidents to be admitted to a hospital with a general admission status as opposed to acute or critical admission status (21.14% compared 16.99%; *χ*^2^(2) = 12.43, *p* = 0.00). Patients in SV accidents were more likely to be cured at discharge compared to those in MV accidents (60.95% compared 56.44%; *χ*^2^(4) = 20.40, *p* = 0.00). Overall, the mean length of stay in-hospital and medical costs associated with their stay were higher for MV patients than patients in SV accidents after tests of normality respectively (Z = −9.15, *p* = 0.00 and Z = −6.91, *p* = 0.00). The average admission period was 21.05 days (SD = 28.52) and the total medical fees were 22,165.80 (SD = 32,946.44) RMB for the total patients.

**Table 2.** Characteristics of patients in SV/MV accident types.

Characteristics	SV (*N* = 1977, %)	MV (*N* = 2119, %)	*χ*^2^/Z	*P*
**Gender**			0.93	0.34
Female	523 (26.45%)	589 (27.80%)		
Male	1,454 (73.55%)	1,530 (72.20%)		
**Age**	33.603±18.466	35.132±14.966	−5.00	0.00
**Month**			16.84	0.12
January	174	168		
February	177	185		
March	172	188		
April	178	145		
May	151	161		
June	128	176		
July	169	185		
August	162	189		
September	152	192		
October	197	183		
November	175	192		
December	142	155		
**Marriage status**			49.84	0.00
Yes	1,060 (53.62%)	1,366 (64.46%)		
No	917 (46.38%)	753 (35.54%)		
**Status in road traffic**			8.84	0.00
Rider	282 (14.26%)	287 (13.54%)		
Pillion	1,222 (61.81%)	1,634 (77.11%)		
**Admission time**			4.75	0.03
Monday to Thursday	1,110 (56.15%)	1,261 (59.51%)		
Friday to Sunday	867 (43.85%)	858 (40.49%)		
Origin*			48.19	0.00
In the district	677 (34.24%)	917 (43.28%)		
Other districts in the city	724 (36.62%)	750 (35.39%)		
Other cities in the province	563 (28.48%)	436 (20.58%)		
Other provinces	13 (0.66%)	16 (0.76%)		
**Admission conditions**			12.43	0.00
Acute	1,557 (78.76%)	1,754 (82.77%)		
Critical	2 (0.10%)	5 (0.24%)		
General	418 (21.14%)	360 (16.99%)		
**Discharge conditions**			20.40	0.00
Cured	1,205 (60.95%)	1,196 (56.44%)		
Get better	646 (32.68%)	795 (37.52%)		
Not cured	40 (2.02%)	23 (1.09%)		
Dead	28 (1.42%)	48 (2.27%)		
Others	58 (2.93%)	57 (2.69%)		
**Rescue in hospital**			0.34	0.56
Yes	1,754 (88.72%)	1,892 (89.29%)		
No	223 (11.28%)	227 (10.71%)		
**Admission number**			0.14	0.71
1 time	1,894 (95.80%)	2,025 (95.56%)		
≥2 times	83 (4.20%)	94 (4.44%)		
Days in hospital	16.56±20.61	25.23±33.77	−9.15	0.00
Total costs in hospital	18,749.35±25,867.64	25,353.3±38,122.70	−6.91	0.00

*The district (county) refers to the district (county) of the hospital in the city.

### 3.3. Comparison between SV and MV accident types regarding injury patterns

As seen in Table 3, the most common body part injured was the head or neck in both SV (*n* = 1,343, 67.93%) and MV (*n* = 1,068, 50.64%) accidents, followed by lower extremities (SV: 280, 14.16%; MV: 594, 28.17%), and the thorax or abdomen (SV: 187, 9.46%; MV: 228, 10.81%). Intracranial injury was the most common injury for patients in SV (*n* = 672, 34.1%) and in MV (*n* = 518, 24.6%) accidents. The second injury pattern for patients in SV accidents was an open head or neck wound (*n* = 261, 13.2%), while for patients in MV accidents it was a fracture to a lower extremity (*n* = 467, 22.1%). A skull fracture and an open wound to the head or neck ranked as the third most common injury among patients involved in SV (*n* = 235, 11.9%) and MV (*n* = 191, 9.1%) accidents. Compared to patients involved in MV accidents, those involved in SV accidents were more likely to sustain a skull fracture (RR: 1.47; 95% CI: 1.22–1.77), an open wound to the head or neck (RR: 1.46; 95% CI: 1.23–1.74), an intracranial injury (RR: 1.39; 95% CI: 1.26–1.53), a head superficial injury (RR: 1.37; 95% CI: 1.01–1.86), an injury to an organ of the thorax or abdomen (RR: 2.0; 95% CI: 1.24–3.26), and a crushing injury to the thorax or abdomen (RR: 1.98; 95% CI: 1.06–3.70). In contrast, individuals involved in SV accidents were less likely than those involved in MV accidents to sustain a spinal fracture (RR: 0.58; 95% CI: 0.39–0.85), a pelvic fracture (RR: 0.22; 95% CI: 0.11–0.46), an upper extremity fracture (RR: 0.75; 95% CI: 0.59–0.96) and injuries to their lower extremities, except for a dislocation, sprain, or injury to a joint or ligament (RR: 0.82; 95% CI: 0.49–1.36).

**Table 3.** Relative risk of different injuries for patients involved in SV and MV accidents.

ICD-10 body region	SV (*N* = 1977, %)	MV (*N* = 2109, %)	RR*	95% CI
**Head or Neck**	1,343 (67.93)	1,068 (50.64)		
Skull fracture	235 (11.89)	171 (8.11)	1.47	1.22-1.77
Intracranial	672 (33.99)	518 (24.56)	1.39	1.26-1.53
Concussion	56 (2.83)	91 (4.31)	0.66	0.47-0.91
Superficial	91 (4.60)	71 (3.37)	1.37	1.01-1.86
Open wound	261 (13.20)	191 (9.06)	1.46	1.23-1.74
Crush	10 (0.51)	15 (0.71)	0.71	0.32-1.58
Neck, nerve injury	18 (0.91)	11 (0.52)	1.75	0.83-3.70
**Thorax or Abdomen**	187 (9.46)	228 (10.81)		
Spinal fracture	39 (1.97)	72 (3.41)	0.58	0.39-0.85
Rib fracture	16 (0.81)	20 (0.95)	0.86	0.45-1.65
Pelvic fracture	9 (0.46)	43 (2.04)	0.22	0.11-0.46
Open wound	14 (0.71)	15 (0.71)	0.99	0.48-2.06
Dislocation, sprain	6 (0.30)	5 (0.24)	1.28	0.39-4.20
Crush	28 (1.42)	15 (0.71)	1.98	1.06-3.70
Organs injury	47 (2.38)	25 (1.19)	2.01	1.24-3.26
Others	28 (1.42)	33 (1.56)	0.91	0.55-1.50
**Upper Extremity**	152 (7.69)	177 (8.39)		
Open wound	13 (0.66)	9 (0.43)	1.55	0.66-3.61
Fracture	102 (5.16)	145 (6.88)	0.75	0.59-0.96
Crush	2 (0.10)	3 (0.14)	0.71	0.12-4.27
Dislocation, sprain, joints, ligaments	11 (0.56)	16 (0.76)	0.74	0.34-1.58
Others	4 (0.20)	4 (0.19)	1.07	0.27-4.27
**Lower Extremity**	280 (14.16)	594 (28.17)		
Open wound	14 (0.71)	43 (2.04)	0.35	0.19-0.64
Fracture	226 (11.43)	467 (22.14)	0.52	0.45-0.60
Crush	9 (0.46)	27 (1.28)	0.36	0.17-0.76
Dislocation, sprain, joints, ligaments	26 (1.32)	34 (1.61)	0.82	0.49-1.36
Others	5 (0.25)	23 (1.09)	0.23	0.09-0.61
**The others**	28 (1.42)	42 (1.99)	0.71	0.44-1.15

*RR: the relative risk is computed for single motorcycle crashes as the reference is multi-vehicle crashes.

## 4. Discussion

Our study retrospectively reviewed the medical records of patients involved in motorcycle injuries to examine differences between SV and MV accident types regarding demographic and clinic characteristics, mortality, and injury patterns.

The greatest of number of patients presented in October, November, February, and March. After the winter months, riders are “brushing the rust” off their motorcycling skills, which is a likely explanation to the accident occurrence observed in February and March. Accidents occurring in October and November may be related to other reasons such as activities' richness during good weather. Savolainen and Mannering showed April and July had 111% and 98% greater probability of being fatal in SV [7]. To a larger extent, the driver bears the sole responsibility for the cause of a SV accident. Driving behavior connected to SV accidents has been a central issue [13],[14], as rider error was the precipitating factor in 66% of SV accidents in the report of Hurt *et al* [1]. This is fact is further testified by the age distribution in SV and MV accident types. The mean age for patients in SV accidents was lower than those in MV accidents. Those of young age are associated with risk taking behaviors and inexperience, which increases their risk of being involved in an accident [15]. Individuals aged greater than 70 years were more likely to suffer from SV accidents. Motorcycle injury accounted for 99.96% of patients. Older motorcyclists are more likely to be involved in SV accidents and sustain severe injuries due to physical character and reduced sensory and perceptual ability [5],[7],[16],[17]. Likewise, they are less likely to sustain from severe or fatal traffic injury in a MV accident.

The average medical costs for patients affected by motorcycle injuries was about US $3,034, while at Vietnam Binh General Hospital, each patient suffering from a motorcycle injury cost on average US $264, or 6 month of average salary, including direct medical costs [18]. The comparing of hospitalization costs should be prudent, as they were affected by many factors. Although we have not detected the factors influencing length of stay in the hospital, Santolino *et al* found that older men with fractures and injuries located in the head and lower torso were more likely to be hospitalized and have a longer expected hospital stay for recovery after accidents [19]. The accident types also determined the length of stay and the costs, except for the numbers of admission and the situation of rescue in-hospital. The explanation to the lack of significance of the two factors maybe that the head injury or internal injuries to the thorax or abdomen in SV accidents counteracted the injury severity of collisions of a motorcycle with a heavy vehicle/bus in MV accidents. The proportion of patients with a general status of admission in SV accidents was higher than those in MV. Patients in MV accidents are more severe than those in SV from the analysis of results of their clinic characteristics, which was in keeping with the result shown by Meuleners *et al* [20]. However, some studies showed the relatively high severity of SV motorcycle accidents [2],[5],[17]. Future study could further compare injury severity through indicators between the two motorcycle accident types [21].

Collision of a motorcycle with a heavier vehicle increased the relative risk of mortality, which is in agreement with the fact that victims in accidents are more easily able to absorb kinetic energy when a motorcycle has a collision with a vehicle having a higher speed and a greater weight [16],[22]–[24]. A study in Brazil showed that collision of a motorcycle with a motorcycle was 11.19 times more likely to result in an injury for a motorcyclist than the collision of a motorcycle with a pedestrian or an animal. Furthermore, those who fell from the motorcycle were 3.81 times more likely to be injured [25]. Early recognition that those victims in MV accidents, especially collisions of motorcycles with heavy vehicles, are more likely to sustain severe injuries than those in SV accidents may mandate corresponding adaptation of clinic staff rescue criteria.

The head was most often the region of principal diagnosis and accounted for 59.11% of injuries, while lower extremity was the second, accounting for 21.43%. Lower-extremity injuries most commonly occur and head injuries have been found to be the greatest cause of fatality in most motorcycle injuries [22],[26]. Although limb injuries are quite common, more than half of all injuries are relatively minor soft tissue injuries, involving only skin and/or subcutaneous tissues [27]. The study population was composed of all “serious injury” patients and those who had light injury in extremities or did not seek medical help were not included. Statistical analysis of the association between injuries in SV following relative risk of injury patterns ranked with thorax or abdomen (RR): organs (2.01), crush (1.98) and head/neck injuries, such as skull (1.47), open (1.47), intracranial (1.39), superficial (1.37). The high incidence of thorax or abdomen injuries followed by the head region is similar to the result a study by Bambach *et al*, which found the thorax had the highest incidence of injury as well as the highest incidence of maximum injury in fatal motorcycle-barrier accidents, followed by the head region [26]. Similarly, it was concluded that patients in MV accidents tend to be vulnerable to injuries of the extremities, especially of the lower extremities, and external trauma to the thorax or abdomen. Furthermore, upon discharge, most patients involved in SV accidents had not been cured when compared to those involved in MV accidents, thus allowing for the deduction that intracranial injury was more serious and more difficult to cure compared with extremity injuries. These results suggest that more attention should be given to head and internal injuries of thorax or abdomen after differentiating the accident type into a SV accident. However, more attention should be given to lower limb injuries and external trauma to thorax or abdomen of MV accidents admitted to the hospital.

Riders and passengers in motorcycle accidents were the primary source of injury (*n* = 3,425, 83.62%), as they can only rely on their protection equipment in case of an accident in China [28]. Helmet use was suggested to be an effective means to reduce the risk of head injury in motorcyclists by nearly 69% and death by nearly 42% [29]. Unfortunately, Li *et al* found helmet use in motorcyclists in the region remained very low [30]. Although data concerning the prevalence of helmet use was not available, wearing a helmet should continue to be mandatory to reduce motorcycle injuries.

As for lower limb injuries, it is easy to presume that extremity injuries will result in some functional limitation and a long recovery time. A descriptive analysis of hospital discharge data among powered two-wheeler users in eight European countries showed lower extremity injuries accounted for 26% of motorcycle injuries and 80% of those injuries were expected to result in some lasting disability. The values corresponding to traumatic brain injury and upper extremity injuries were 19% to 24% and 21% to 47%, respectively [31]. Therefore, the importance of protecting extremities, especially the lower limb, must be stressed. Otte *et al* reported that protective gear and high boots significantly reduced the incidence of limb injuries and complex leg fractures [32]. In contrast, Lin and Kraus concluded that there was limited evidence to support the effect of protective boots, jackets, leg protectors, etc., to motorcycle safety [33]. Internal injuries of the thorax or abdominal region were found to be associated with SV accidents, while external traumas resulting in pelvic fracture and spinal fracture were found to be connected with MV accidents. This suggests that protecting the thorax and abdominal region should be very important, no matter whether the accident type is SV or MV. Protective clothing could reduce mortality by protecting thorax and abdominal part [34],[35]. Protective clothing in conjunction with other accessories are rarely used in the region, a fact which is common in other areas in China. To date, there are no suitable protective clothing mandates, nor any mandatory requirements for using motorcycle accessories [27]. At the same time, mandating the use of protective clothing is not recommended [35]. Thus, educational prevention and enforcement of laws for helmet use to reduce motorcycle injuries are relied upon at present.

There are some limitations in this study. First, data of on deaths occurring at the accident scene and after hospitalization could not be collected. Therefore, the mortality and injury patterns described here are large underestimations of the absolute mortality risks associated with accident types. Secondly, a possible bias might have been introduced by coding. Thirdly, this study presents an analysis of a major comprehensive hospital in a city concerning the comparison of hospitalized motorcycle injury victims in SV and MV accident types. Therefore, cautiousness is necessary when the conclusions are extended.

## 5. Conclusion

The relative risk for death for patients in MV is higher than in SV, especially in collision with a mass vehicle(s). Head injury is the dominate form of serious motorcycle injury. SV accidents have a higher correlation with head injury or internal injuries to the thorax or abdomen, while MV accidents have a higher correlation with extremity injuries, especially to the lower extremities, or external trauma to the thorax or abdomen. Enforcement of laws for helmet use is the most likely effective prevention method for the reduction of motorcycle injuries.
